# Benefits of an accelerated climatotherapy programme for busy people: comparisons according to area and season

**DOI:** 10.1007/s00484-023-02595-0

**Published:** 2023-12-13

**Authors:** Hitomi Kanayama, Yukinori Kusaka, Hiroyuki Inoue, Takayoshi Hirai, Yuko Agishi, Angela Schuh

**Affiliations:** 1 Division of Environmental Health, Department of International Social and Health Sciences, Faculty of Medical Sciences, University of Fukui, Eiheiji, Japan; 2 Research Institute of Health Resort Medicine, Tokyo, Japan; 3 University of Fukui, Fukui, Japan; 4 Division of Generic and Global Studies, Faculty of Education, Humanities and Social Sciences, University of Fukui, Fukui, Japan; 5 Faculty of Nursing and Social Welfare Sciences, Fukui Prefectural University, Eiheiji, Japan; 6 Hokkaido University, Sapporo, Japan; 7 Ludwig-Maximilians-University Munich, Munich, Germany

**Keywords:** Climatotherapy programme, Forest environment, Mountain, Intervention, Health promotion, Busy people

## Abstract

An accelerated climatotherapy programme was evaluated for use with busy people in mid-mountain and flat lowland areas. A total of 43 urban residents participated in this climatotherapy programme. Participants’ blood pressure, pulse rate, peripheral skin temperature and levels of salivary amylase, salivary cortisol and blood lactate were measured, and they completed the Profile of Mood Status questionnaire. In the mid-mountain area, which had a cooler environment and long uphill paths, participants’ percentage of maximum pulse rate (70.01%) to estimated maximum heart rate was higher than that (59.67%) of participants in the flat lowland area, suggesting that the mid-mountain area was suitable for endurance training. At both sites, the decrease in peripheral skin temperature during the climatic terrain cure suggested that our programme was properly implemented with a cool body surface in accordance with our purpose. Negative moods improved quickly, suggesting that the forest environment and the fresh-air rest cure may have relaxed participants. In late spring and early autumn, the mood of approximately 25% of participants improved to an Iceberg profile, which is associated with positive mental states and athletic peak performance, after climatotherapy. On the other hand, the weather in early spring and late autumn was more likely to facilitate maintenance of a cool body surface during the climatic terrain cure. With the support of individualized feedback provided after the climatotherapy sessions, three participants developed regular exercise habits, serving as a good example of the effectiveness of our climatotherapy programme to elicit behavioural change.

## Introduction

Climatotherapy is conducted in uplands, mountains and seaside areas and consists of several weeks of exposure to suitable biometeorological conditions (Schuh 1993). A controlled study implemented 3 weeks of climatotherapy and assessed blood lactate levels under bicycle ergometer loading; the cool condition group exhibited significantly lower blood lactate levels than the thermally neutral group. And a cool body surface improved aerobic capacity when walking (Schuh 1991). The long-term efficacy of modern climatotherapy has been documented and validated according to evidence-based medicine (Schuh 2017).

Considering the human circaseptan rhythm, individuals are recommended to spend at least 3 weeks at a health resort to experience a therapeutic effect (Amelung and Hildebrandt 1986). However, the Japanese population spends the least time on leisure activities among major Western countries (Flath 2014). We adapted the climatotherapy method focusing on a cool body surface developed at the University of Munich and designed an accelerated climatotherapy programme for time-pressed people in Japan.

In our pilot study, systolic blood pressure (SBP) significantly decreased, and pulse rate (PR) significantly increased during the climatic terrain cure on the mid-mountain path. On the lowland flat path, blood pressure (BP) was not significantly altered. At both sites, the Profile of Mood States (POMS) scores indicated significant improvement after the half-day climatotherapy sessions, with the exception of the Vigour subscale (Kanayama et al. 2017).

In the current study, we administered our climatotherapy programme to urban residents in two forest environments, namely, a mid-mountain area and a flat lowland area (control area). We designed the programme to encourage spontaneous behaviour change in participants by giving them immediate access to physical and psychological measurement results throughout their participation in the programme. For this reason, small instruments were chosen that are easy to use in the field and provide immediate results. To increase the effectiveness of the programme, one of the authors (HK) also posted a report to each participant after three sessions in the spring and autumn, with health promotion advice based on the data collected.

By increasing the sample size from that in the short report (Kanayama et al. 2017) and by increasing the number of measures in addition to BP, PR and POMS, we aimed to (1) identify site- and season-specific differences in changes in blood pressure and pulse rate, skin temperature and salivary amylase during climatotherapy sessions; (2) identify site-specific differences in changes in blood lactate levels before and after climatic terrain cure; (3) examine changes in POMS *T*-score before and after climatotherapy sessions for the six mood subscales; and (4) determine the extent to which the three spring climatotherapy sessions and individual feedback and advice after the sessions led to behavioural changes in participants.

## Materials and methods

### Study design

The current study was a prospective interventional study with climatotherapy programme before-after trials.

### Participants

Participants were recruited from Fukui city and its suburbs, which were not mountain or forest environments. Our eligibility criteria were people with no mobility problems and a desire to improve their health. Of these, we considered physical inactivity as the most suitable indication. Contraindications were acute phase of infection, acute phase of myocardial infarction, acute phase of cerebral infarction, poorly controlled diabetes, grade III hypertension, NYHA III and IV heart failure, mitral stenosis, Fontaine classification III and IV degree arteriosclerosis obliterans, cor pulmonale with increased pulmonary artery pressure, skin diseases such as systemic lupus erythematosus induced or aggravated by sunlight exposure, rheumatic diseases, systemic sclerosis and mental diseases and alcoholism. Subjects from the pilot study also participated in the current study, with the exception of subjects from 2014.

### Setting and locations

Our climatotherapy sites were in Fukui prefecture, located in central Japan on the Sea of Japan side. In the current study, we administered our climatotherapy programme in two forest environments, namely, a mid-mountain area (Yatsusugi Forest [YF]) and a flat lowland area as a control area (Fukui Prefectural General Green Center [GC]). In the spring and autumn, when the participants’ data were collected, these areas belong to a generally protective climate with a wide range of indications. On the other hand, the rainy season and summer are hot and humid, with a loading climate that is not suitable for climatotherapy. The winter season is cold and snowy, with a stimulating climate, and climatotherapy can only be carried out for training purposes.

**Fig. 1:**
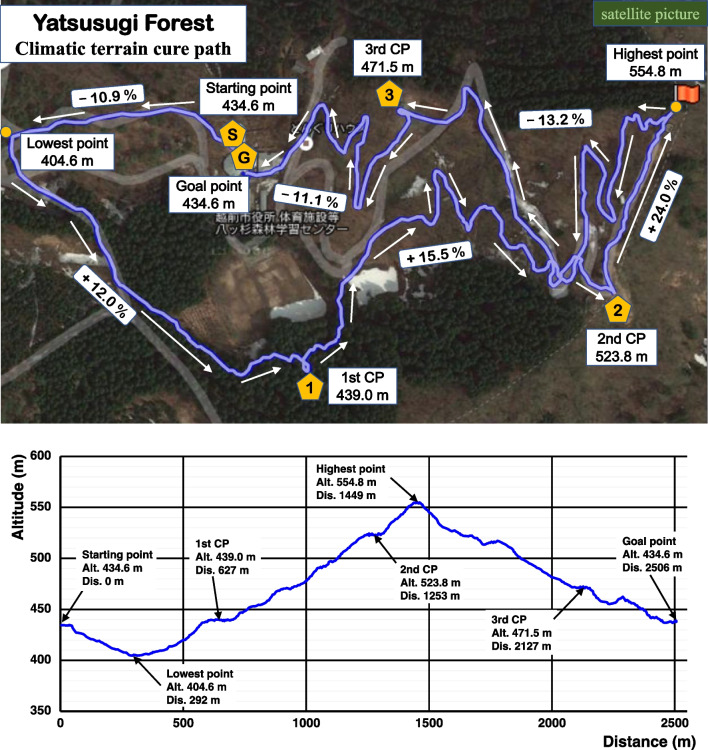
The path used for the climatic terrain cure in Yatsusugi Forest, a mid-mountain area. Alt., altitude; Dis., distance; CP, checkpoint

Time-series location data of paths were obtained by a GPS data logger device (i-gotU GT-820Pro, Mobile Action Technology, Inc., Taiwan), and a topographic map was drawn based on time-series latitude, longitude and altitude data. The path in YF used for the climatic terrain cure included several uphill and downhill portions. The total length of the path was 2506 m, and the altitudes of highest and lowest points were 554.8 m and 404.6 m, respectively (Fig. 1). In contrast, the GC path was almost flat. The total length of the path was 2247 m, and the altitudes of the highest and lowest points were 45.2 m and 36.7 m, respectively. Both the YF and GC paths were in tree-rich environments, alternating between sun-exposed paths without tree cover and shaded paths with tree cover.

### Interventions

**Fig. 2:**
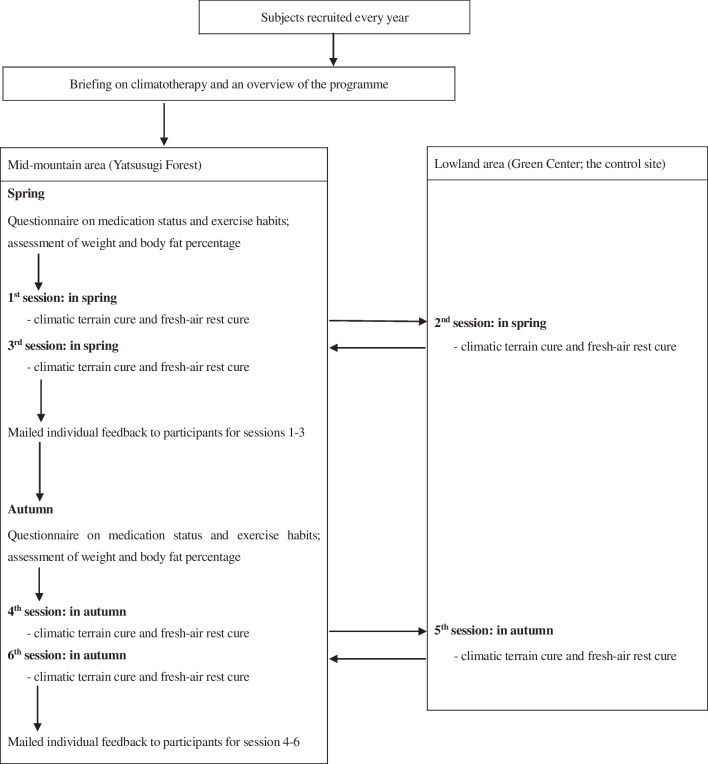
Flowchart of the current study

Our programme was conducted in spring and autumn of 2015–2017 (Fig. 2). A 1-week interval served as a washout period between each of the three sessions in both spring and autumn.

In the spring, before the start of the programme, all participants were given an overview of our climatotherapy programme and gave written informed consent. At the beginning of the first and fourth sessions, all participants completed questionnaires about their medication status and exercise habits and were body measured. All participants were forbidden to exercise and to drink alcohol from the previous evening until the morning of all sessions. In all six sessions, which were a half day in length and started in the morning, all participants underwent a climatic terrain cure and a 20-min fresh-air rest cure under ‘slightly cool’ conditions. The fresh-air rest cure involved resting in the supine position in the shade while exposed to the open air.

The participants’ walking pace was guided and controlled by a qualified climatotherapist (HK). The standard pace during the climatic terrain cure was set at 80 steps per minute on the uphill and 90 steps per minute on the flat or downhill, with a metronome sounding to maintain the pace. The target and risk PRs of the participants during the climatic terrain cure were set 180 − age beats per minute (bpm) and 220 − age bpm with exercise habits and 160 − age bpm and 200 − age bpm without exercise habits. For participants aged 65 years or older or taking antihypertensive drugs such as beta-blockers that could cause bradycardia, target and risk PRs were 10% lower.

At each checkpoint, the participants’ blood pressure, pulse rate, skin temperature and subjective the American Society of Heating Refrigerating and Air-Conditioning Engineers (ASHRAE) scale were checked to ensure that they were within an effective and safe range. ASHRAE scale was developed to quantify an individual’s thermal sensation defined as follows: cold (− 3), cool (− 2), slightly cool (− 1), indifferent (0), slightly warm (1), warm (2) and hot (3). If necessary, walking pace was changed, and participants were asked to maintain a ‘slightly cool’ subjective temperature (ASHRAE scale =  − 1) be removing their clothes or rolling up their sleeves.

Data collection was carried out, and all participants received individual feedback according to the schedule shown in Fig. 2. These feedbacks include advice on healthier lifestyle habits, such as the intensity and frequency of exercise and appropriate sun exposure for them.

### Primary and secondary outcome measures

#### Meteorological data collection

On-site meteorological data (temperature, relative humidity and wind speed) were collected with a portable weather tracker (Kestrel 4000 Weather Meter, KestrelMeters.com, USA). To prevent equipment from being ruined by rain or being stolen, a portable weather tracker was brought to the study site on each session day to collect on-site data. Therefore, it was not possible to collect weather data at both YF and GC on the same day. For comparison, meteorological data observed at the Japan Meteorological Agency (JMA) Fukui local meteorological office were obtained from the JMA website (Japan Meteorological Agency 2023) for the dates and times when the climatotherapy sessions were conducted.

#### Body measurements

Participants’ height data were obtained with their consent from reports of health checkups carried out in their workplace or community. Body weight and body fat percentage were measured using a body composition analyser (HBF-214, Omron, Japan). The measurements were carried out twice, once before the spring sessions and once before the autumn sessions.

Body mass index (BMI) was calculated as body weight (kg) divided by height (m) squared.

#### Blood pressure and pulse rate

Systolic blood pressure (SBP), diastolic blood pressure (DBP) and PR were measured with portable automatic blood pressure and pulse rate monitors (HEM-6320, Omron, Japan). Before the start and after the sessions, BP and PR were measured at rest in a sitting position; at other points during the climatic terrain cure, these variables were measured in a standing position immediately after arrival at the measurement site. A total of six measurements were taken in each session: before the session (starting point), four times during the climatic terrain cure (1st checkpoint, 2nd checkpoint, 3rd checkpoint and goal point), and after the session (after the fresh-air rest cure).

In addition, maximum heart rate (HR_max_) was estimated according to the age of each participant using the Gellish formula (Gellish et al. 2007), which is applicable to adults of a wide range of ages and fitness levels. Percent PR_max_, the highest percentage of PR to estimated HR_max_ achieved during the session, was also calculated for each participant in each of the six sessions.

#### Peripheral skin temperature and subjective temperature

The central dorsal area of the hand was chosen as the measurement position to assess peripheral skin temperature, as it was not covered by clothing and was easy to measure. Skin temperature was measured with an infrared thermometer (AD5614, A&D Company, Limited, Japan). In each session, peripheral skin temperature measurements were taken a total of six times at the same time as BP and PR measurements. In addition to these measurements, participants responded to their subjective temperature sensations on the ASHRAE scale.

#### Salivary amylase levels

Participants were instructed not to eat, including sweets, smoke or brush their teeth for 1 h prior to collecting the saliva sample. In each session, saliva samples from participants were collected at the sublingual position with disposable test strips before the session (starting point), after the climatic terrain cure (goal point) and after the session (after the fresh-air rest cure). Salivary amylase (sAMY) activity levels were automatically quantified with a dry chemistry system ‘salivary amylase monitor’ (NIPRO, Japan).

#### Salivary cortisol levels

Participants were also instructed not to eat, including sweets, smoke or brush their teeth for 1 h prior to collecting the saliva sample. In 2016 and 2017, saliva samples were collected with Salivette® systems before and after the sessions and were frozen and immediately sent to a laboratory testing company (SRL, Inc., Japan) for examination.

#### Blood lactate levels

Blood lactate levels were measured by Lactate Pro 2 (Arkray, Japan) (Pyne et al. 2000) and a Lactate Pro 2 sensor. In each session, before and after the climatic terrain cure (at the starting point and goal point), a small amount of peripheral blood was collected from participants by fingertip puncture. Blood lactate measurements were performed to ensure that after the climatic terrain cure did not exceed lactate levels of 4 mmol/L, the Onset of Blood Lactate Accumulation (OBLA).

#### Questionnaires

Participants completed a questionnaire twice, once before the spring sessions and once before the autumn sessions, on their current health status, medication status and exercise habits. Questions on medication status and regular exercise habits were taken from the National Health and Nutrition Survey (NHNS) questionnaire (Ministry of Health, Labour and Welfare 2023). The NHNS survey defines regular exercise habits as exercising at least 2 days a week, at least 30 min a day, over at least a year.

To assess participants’ subjective mood, the brief form of the POMS, Japanese edition (Yokoyama et al. 1990), was used. This form is comprised of 30 items and yields six subscales: Tension-Anxiety (T-A), Depression-Dejection (D), Anger-Hostility (A-H), Vigour (V), Fatigue (F) and Confusion (C). Scores on each subscale before and after the session were compared using *T*-scores standardized by sex and age. In each session, participants answered this form before the session about their average mood over the previous week and after the session about their mood at that time.

### Statistical analysis

The sample size was calculated by a priori power analysis using G*Power 3 (Faul et al. 2007). The *α* level was set to 0.05, 1-β was set to 0.80 and the effect size was set to 0.50 for a paired *t* test and 0.20 for a one-way analysis of variance (ANOVA). A paired *t* test was used for group comparisons, and a two-tailed *p* value less than 0.05 was considered significant. For analysis of each variable, only data from participants with no missing values were included. For variables measured three or more times during the session, a one-way repeated measures ANOVA was performed, and multiple comparisons were conducted using Dunnette’s method. The statistical analyses were conducted with SPSS v28.0 (IBM Japan, Ltd., Japan).

## Results

### Characteristics of participants

Table 1 shows the participants’ characteristics and body measurement data obtained before first (spring) and fourth (autumn) sessions. Excluding one participant who dropped out due to family reasons, a total of 43 participants (20 men and 23 women) from 2015 to 2017 were evaluated. The mean height, weight, BMI and body fat percentage of participants by sex were highly similar to the NHNS results in 2015–2017. The proportion of participants who exercised regularly was much higher among those aged 60 and over, and much lower among those under 60.

**Table 1:** Demographic and body measurement data before the first (spring) and fourth (autumn) sessions (*n* = 43)

	Spring		*p* value*	Autumn		*p* value*
Sex, *n* (%)						
Male	20 (46.5%)			20 (46.5%)		
Female	23 (53.5%)			23 (53.5%)		
Age, years (mean ± SD)	65.7 ± 10.1			66.3 ± 10.1		
Male	67.0 ± 12.8			67.6 ± 12.8		
Female	64.7 ± 7.3		*p* = 0.473	65.2 ± 7.3		*p* = 0.440
Regular exercise habits, *n* (%)	Yes	No		Yes	No	
All participants	28 (65.1%)	15 (34.9%)		29 (67.4%)	14 (32.6%)	
Males	12 (60.0%)	8 (40.0%)		13 (65.0%)	7 (35.0%)	
Females	16 (69.6%)	7 (30.4%)	*p* = 0.540	16 (69.6%)	7 (30.4%)	*p* = 1.000
< 60 years	1 (12.5%)	7 (87.5%)		1 (12.5%)	7 (87.5%)	
60–79 years	27 (77.1%)	8 (22.9%)	*p* = 0.001	28 (80.0%)	7 (20.0%)	*p* = 0.001
Medication status	Yes	No		Yes	No	
All participants	19 (44.2%)	24 (55.8%)		21 (48.8%)	22 (51.2%)	
Males	11 (55.0%)	9 (45.0%)		12 (60.0%)	8 (40.0%)	
Females	8 (34.8%)	15 (65.2%)	*p* = 0.228	9 (39.1%)	14 (60.9%)	*p* = 0.227
< 60 years	0 (0.0%)	8 (100.0%)		0 (0.0%)	8 (100.0%)	
60–79 years	19 (54.3%)	16 (45.7%)	*p* = 0.006	21 (60.0%)	14 (40.0%)	*p* = 0.004
Antihypertensive drugs	14 (32.6%)	29 (67.4%)		15 (34.9%)	28 (65.1%)	
Anti-arrhythmic drugs	0 (0.0%)	43 (100.0%)		0 (0.0%)	43 (100.0%)	
Antidiabetic drugs	4 (9.3%)	39 (90.7%)		3 (7.0%)	40 (93.0%)	
Cholesterol-lowering drugs	10 (23.3%)	33 (76.7%)		12 (27.9%)	31 (72.1%)	
Anti-hyperlipidaemia drugs	2 (4.7%)	41 (95.3%)		1 (2.3%)	42 (97.7%)	
Anti-anaemic agents (iron pill)	0 (0.0%)	43 (100.0%)		0 (0.0%)	43 (100.0%)	
Height, cm (mean ± SD)						
Males	165.5 ± 5.9					
Females	155.5 ± 4.6		*p* < 0.001			
Weight, kg (mean ± SD)						
Males	64.8 ± 10.4			64.2 ± 10.3		*p* = 0.049
Females	53.6 ± 6.8			53.7 ± 7.1		*p* = 0.829
BMI, kg/m^2^ (mean ± SD)						
Males	23.6 ± 2.8			23.4 ± 2.8		*p* = 0.052
Females	22.1 ± 2.4			22.2 ± 2.5		*p* = 0.905
Body fat, % (mean ± SD)						
Males	25.3 ± 3.3			25.7 ± 3.0		*p* = 0.389
Females	32.8 ± 3.3			32.6 ± 3.7		*p* = 0.679

^*^p values derived from *t* tests or Fisher’s exact tests

*SD* standard deviation, *BMI* body mass index

### Primary and secondary outcome measures

#### Meteorological data

Weather data at the study sites (YF and GC) and at the Fukui city centre (JMA Fukui) are shown in Tables 2 and 3.

**Table 2:** Comparison of the average of site-specific meteorological observations during the climatic terrain cure with the average of JMA Fukui data in 2015–2017†

Site	Meteorological factor	On-site data	JMA Fukui data	*t*	*p* value	Effect size (Cohen’s *d*)
YF	Temperature (°C)	18.47 ± 3.94	21.49 ± 3.88	− 15.777	< 0.001	− 0.772
	Relative humidity (%)	70.42 ± 15.05	55.56 ± 13.31	17.801	< 0.001	1.019
	Wind speed (m/s)	0.68 ± 0.53	3.09 ± 2.22	− 9.483	< 0.001	− 1.422
GC	Temperature (°C)	21.51 ± 3.69	21.18 ± 3.39	1.974	0.055	0.090
	Relative humidity (%)	57.59 ± 14.31	53.20 ± 13.34	6.349	< 0.001	0.310
	Wind speed (m/s)	0.60 ± 0.43	3.93 ± 1.67	− 13.288	< 0.001	− 2.606

JMA Fukui is located at 8.8 m above sea level

^†^All values are presented as the mean ± standard deviation

*JMA* Japan Meteorological Agency, *SD* standard deviation

**Table 3:** Average of on-site meteorological observations at YF and GC during sessions in 2015–2017†

		Spring			Autumn		
Site	Meteorological factor	1st session	2nd session	3rd session	4th session	5th session	6th session
YF	Temperature (°C)	20.26 ± 1.62	––-	21.69 ± 2.60	19.29 ± 3.81	––-	16.28 ± 2.93
	Relative humidity (%)	47.25 ± 7.12	––-	68.19 ± 6.14	71.73 ± 4.63	––-	77.05 ± 14.06
	Wind speed (m/s)	0.60 ± 0.62	––-	0.60 ± 0.42	0.87 ± 0.68	––-	0.47 ± 0.45
GC	Temperature (°C)	––-	23.61 ± 3.69	––-	––-	22.12 ± 3.85	––-
	Relative humidity (%)	––-	47.21 ± 14.32	––-	––-	57.45 ± 4.59	––-
	Wind speed (m/s)	––-	0.75 ± 0.46	––-	––-	0.59 ± 0.39	––-

^†^All values are presented as mean ± standard deviation

As both YF and GC were in tree-rich environments, these locations were significantly more humid and less windy than the JMA Fukui site, which was in an urban environment. The mean temperatures at YF were significantly lower than those at JMA Fukui, but those at GC did not significantly differ from those at JMA Fukui.

#### Body measurements

There were no significant changes except for body weight in men.

#### Blood pressure and pulse rate

SBP, DBP and PR data obtained at each measurement point in YF and GC and the results of one-way repeated measures ANOVAs with multiple comparisons conducted using Dunnette’s method are shown in Table 4.

**Table 4:** SBP, DBP and PR at each CP at YF and GC (*n* = 43)†

	Season	Session	Site	Starting point	1st CP	2nd CP	3rd CP	Goal point	After fresh-air rest cure	Effect size (*η*_p_^2^)
SBP (mmHg)	Spring	1	YF	141.3 ± 20.2	150.4 ± 25.4 *p* = 0.011	131.7 ± 22.9 *p* = 0.007	120.3 ± 20.8 *p* < 0.001	121.2 ± 19.4 *p* < 0.001	133.9 ± 19.6 *p* = 0.061	0.418
		2	GC	134.3 ± 16.6	131.1 ± 20.4 *p* = 0.449	127.2 ± 20.7 *p* = 0.006	125.7 ± 17.7 *p* < 0.001	124.8 ± 16.7 *p* < 0.001	127.6 ± 19.5 *p* = 0.010	0.115
		3	YF	131.9 ± 19.2	148.0 ± 24.4 *p* < 0.001	130.4 ± 27.7 *p* = 0.984	117.2 ± 19.3 *p* < 0.001	117.6 ± 19.6 *p* < 0.001	127.0 ± 17.2 *p* = 0.329	0.407
	Autumn	4	YF	134.5 ± 20.7	150.2 ± 24.5 *p* < 0.001	134.7 ± 20.2 *p* = 1.000	122.7 ± 23.3 *p* = 0.004	119.7 ± 23.3 *p* < 0.001	127.1 ± 18.3 *p* = 0.132	0.323
		5	GC	133.9 ± 18.0	132.3 ± 20.3 *p* = 0.946	126.3 ± 21.4 *p* = 0.009	124.0 ± 20.1 *p* < 0.001	122.5 ± 21.3 *p* < 0.001	127.9 ± 17.1 *p* = 0.057	0.142
		6	YF	134.8 ± 20.2	153.3 ± 29.4 *p* < 0.001	134.5 ± 26.5 *p* = 1.000	120.0 ± 19.9 *p* < 0.001	122.3 ± 20.2 *p* < 0.001	132.0 ± 19.3 *p* = 0.803	0.431
DBP (mmHg)	Spring	1	YF	82.1 ± 11.1	87.4 ± 15.6 *p* = 0.063	75.4 ± 16.3 *p* = 0.009	74.6 ± 12.7 *p* = 0.003	73.9 ± 12.5 *p* < 0.001	81.7 ± 11.4 *p* = 1.000	0.229
		2	GC	78.2 ± 11.3	77.7 ± 13.3 *p* = 0.997	75.4 ± 14.6 *p* = 0.261	74.2 ± 10.1 *p* = 0.042	72.5 ± 10.9 *p* = 0.002	77.6 ± 10.6 *p* = 0.995	0.093
		3	YF	78.2 ± 10.9	82.5 ± 12.8 *p* = 0.052	73.5 ± 12.2 *p* = 0.032	70.3 ± 10.4 *p* < 0.001	69.4 ± 12.3 *p* < 0.001	78.9 ± 9.0 *p* = 0.993	0.307
	Autumn	4	YF	79.3 ± 10.6	82.3 ± 15.0 *p* = 0.570	76.2 ± 12.4 *p* = 0.541	71.3 ± 14.9 *p* = 0.003	70.5 ± 12.4 *p* = 0.001	79.0 ± 15.7 *p* = 1.000	0.161
		5	GC	77.8 ± 12.1	76.2 ± 13.4 *p* = 0.812	72.3 ± 12.5 *p* = 0.007	71.3 ± 13.6 *p* < 0.001	70.6 ± 12.1 *p* < 0.001	77.0 ± 13.1 *p* = 0.989	0.139
		6	YF	79.7 ± 13.5	84.3 ± 12.6 *p* = 0.110	74.6 ± 15.2 *p* = 0.051	72.4 ± 14.3 *p* = 0.002	72.1 ± 12.6 *p* = 0.001	81.1 ± 10.9 *p* = 0.943	0.225
PR (beats/min)	Spring	1	YF	70.4 ± 10.0	90.6 ± 17.4 *p* < 0.001	104.2 ± 19.3 *p* < 0.001	100.6 ± 15.6 *p* < 0.001	97.6 ± 15.8 *p* < 0.001	73.8 ± 10.8 *p* = 0.254	0.726
		2	GC	73.5 ± 13.0	85.4 ± 14.2 *p* < 0.001	87.7 ± 15.3 *p* < 0.001	88.3 ± 14.5 *p* < 0.001	90.3 ± 16.8 *p* < 0.001	71.0 ± 11.5 *p* < 0.001	0.666
		3	YF	72.3 ± 12.2	90.0 ± 14.8 *p* < 0.001	107.1 ± 19.1 *p* < 0.001	97.4 ± 15.6 *p* < 0.001	93.6 ± 17.7 *p* < 0.001	72.3 ± 11.9 *p* = 1.000	0.769
	Autumn	4	YF	68.9 ± 12.3	93.9 ± 21.1 *p* < 0.001	112.1 ± 21.5 *p* < 0.001	101.8 ± 17.3 *p* < 0.001	99.6 ± 17.3 *p* < 0.001	72.7 ± 12.4 *p* = 0.198	0.775
		5	GC	67.1 ± 10.5	88.9 ± 17.2 *p* < 0.001	94.8 ± 15.5 *p* < 0.001	93.9 ± 17.2 *p* < 0.001	94.0 ± 18.5 *p* < 0.001	71.8 ± 12.2 *p* = 0.014	0.747
		6	YF	70.9 ± 10.9	99.7 ± 19.5 *p* < 0.001	114.3 ± 19.0 *p* < 0.001	102.4 ± 15.7 *p* < 0.001	100.2 ± 16.6 *p* < 0.001	72.1 ± 12.6 *p* = 0.961	0.817

A one-way repeated measures analysis of variance was performed, and multiple comparisons were made using Dunnette’s method. The reference category was the starting point

^†^All values are presented as the mean ± standard deviation

*SBP* systolic blood pressure, *DBP* diastolic blood pressure, *PR* pulse rate, *SD* standard deviation, *CP* checkpoint

In all sessions at YF, SBP at the 1st CP was significantly higher than that at the starting point and continued to decrease from the 1st CP to the goal point. After the fresh-air rest cure, SBP was higher than at the goal point but lower than at the starting point. However, there was no significant difference in SBP between at the starting point and after the fresh-air rest cure in any session.

At GC, SBP during the session was lower than at the starting point.

At both YF and GC, the DBP dynamics followed a similar pattern as the SBP dynamics.

At both YF and GC, PR during the climatic terrain cure was significantly higher than that at the starting point but returned to the starting point level after 20 min of fresh-air rest cure.

Percent PR_max_ was calculated for each of the 39 participants in each of the six sessions, except for four participants who took antihypertensive drugs that may cause bradycardia. The average percent PR_max_ in both spring and autumn was 70.01% at YF and 59.67% at GC.

#### Peripheral skin temperature

The peripheral skin temperature during the climatotherapy sessions at YF and GC and the results of the one-way repeated measures ANOVA and multiple comparisons using Dunnette’s method are shown in Table 5. At both YF and GC, peripheral skin temperatures decreased significantly during climatic terrain cure. In all sessions, peripheral skin temperature was higher after the fresh-air rest cure than at the goal point.

**Table 5:** Peripheral skin temperature during climatotherapy at YF and GC (*n* = 43)

	Season	Session	Site	Starting point	1st CP	2nd CP	3rd CP	Goal point	After the fresh-air rest cure	Effect size (*η*_p_^2^)
Skin T (°C)	Spring	1	YF	28.5 ± 2.4	26.3 ± 1.8 *p* < 0.001	27.2 ± 1.8 *p* < 0.001	28.6 ± 1.7 *p* = 0.987	29.0 ± 1.9 *p* = 0.263	30.4 ± 1.7 *p* < 0.001	0.517
		2	GC	31.7 ± 1.7	29.3 ± 2.7 *p* < 0.001	28.8 ± 3.0 *p* < 0.001	28.8 ± 2.4 *p* < 0.001	28.3 ± 2.8 *p* < 0.001	30.0 ± 2.7 *p* < 0.001	0.395
		3	YF	31.3 ± 1.5	27.5 ± 2.1 *p* < 0.001	28.7 ± 2.3 *p* < 0.001	28.8 ± 1.8 *p* < 0.001	29.1 ± 1.8 *p* < 0.001	31.4 ± 2.3 *p* = 0.996	0.535
	Autumn	4	YF	29.7 ± 2.9	26.3 ± 2.3 *p* < 0.001	25.4 ± 3.0 *p* < 0.001	26.5 ± 3.3 *p* < 0.001	26.7 ± 2.7 *p* < 0.001	29.3 ± 3.2 *p* = 0.588	0.530
		5	GC	31.2 ± 2.5	28.5 ± 2.6 *p* < 0.001	28.0 ± 2.5 *p* < 0.001	28.1 ± 2.9 *p* < 0.001	27.0 ± 2.9 *p* < 0.001	28.9 ± 3.1 *p* < 0.001	0.552
		6	YF	30.0 ± 2.2	25.4 ± 2.2 *p* < 0.001	24.3 ± 3.4 *p* < 0.001	26.2 ± 3.4 *p* < 0.001	26.0 ± 3.4 *p* < 0.001	27.7 ± 3.4 *p* < 0.001	0.469

Peripheral skin temperature values are presented as the mean ± standard deviation. A one-way repeated measures analysis of variance was conducted, and multiple comparisons were made using Dunnette’s method. The reference category was the starting point

*Skin T* skin temperature, *SD* standard deviation, *CP* checkpoint

#### Salivary amylase levels

sAMY activity levels during the climatotherapy sessions at YF and GC and the results of the one-way repeated measures ANOVA and multiple comparisons using Dunnette’s method are shown in Table 6. However, sAMY levels varied widely, with a large standard deviation (SD) across sessions.

**Table 6:** Salivary amylase activity levels at YF and GC (*n* = 43)

	Season	Session	Site	Starting point	After the climatic terrain cure	After the fresh-air rest cure	Effect size (*η*_p_^2^)
sAMY activity level (KU/L)	Spring	1	YF	81.1 ± 73.9	71.6 ± 114.5 *p* = 0.743	65.2 ± 73.4 *p* = 0.453	0.014
		2	GC	65.5 ± 77.9	57.9 ± 79.6 *p* = 0.762	33.3 ± 30.9 *p* = 0.020	0.082
		3	YF	65.5 ± 85.4	82.3 ± 89.2 *p* = 0.346	67.9 ± 76.1 *p* = 0.976	0.022
	Autumn	4	YF	97.7 ± 145.9	67.6 ± 90.3 *p* = 0.206	58.1 ± 60.7 *p* = 0.074	0.053
		5	GC	79.4 ± 106.3	80.0 ± 65.3 *p* = 0.999	59.3 ± 64.5 *p* = 0.332	0.027
		6	YF	93.7 ± 91.9	101.6 ± 124.5 *p* = 0.878	60.8 ± 67.3 *p* = 0.148	0.059

Salivary amylase activity levels are indicated as the mean ± standard deviation. A one-way repeated measures analysis of variance was conducted, and multiple comparisons were made using Dunnette’s method. The reference category was the starting point

*sAMY* salivary amylase, *SD* standard deviation

#### Salivary cortisol levels

Data are not shown. Results of these analyses will be provided in a separate short communication.

#### Blood lactate levels

Blood lactate levels were assessed before and after the climatic terrain cure at YF and GC, and the results of the paired *t* tests are shown in Table 7. After the climatic terrain cure, blood lactate levels increased at YF but decreased slightly at GC. Furthermore, in both spring and autumn, the increase in blood lactate levels at YF was greater in the first session of the season than in the last session of the season. However, no significant changes in blood lactate levels were observed between before and after the climatic terrain cure, except for the first autumn session at YF, where blood lactate levels increased significantly.

**Table 7:** Blood lactate levels before and after the climatic terrain cure at YF and GC (*n* = 43)

	Season	Session	Site	Starting point	After the climatic terrain cure	*t*	*p* value	Effect size (Cohen’s *d*)
Blood lactate level (mmol/L)	Spring	1	YF	2.45 (1.92, 2.97)	3.03 (2.38, 3.68)	− 1.449	0.155	− 0.304
		2	GC	1.88 (1.51, 2.26)	1.70 (1.48, 1.91)	0.875	0.386	0.185
		3	YF	2.26 (1.84, 2.68)	2.48 (2.02, 2.94)	− 0.913	0.366	− 0.156
	Autumn	4	YF	2.07 (1.72, 2.43)	2.76 (2.20, 3.32)	− 2.203	0.033	− 0.446
		5	GC	2.10 (1.77, 2.43)	2.02 (1.67, 2.37)	0.350	0.728	0.072
		6	YF	2.20 (1.82, 2.58)	2.48 (2.08, 2.88)	− 1.103	0.276	− 0.227

Blood lactate levels are indicated as the mean (95% CI)

*CI* confidence interval

#### Health-related behavioural changes

We compared the exercise habits of the subjects before the first spring session and before the first autumn session, finding that two female participants ceased their regular exercise, while three subjects—one couple and one female—started exercising regularly.

#### POMS scores

The POMS *T*-scores on the six mood subscales before and after climatotherapy sessions at YF and GC and the results of the paired *t* tests are shown in Table 8. In all 6 sessions, *T*-scores for the five negative mood subscales (T-A, D, A-H, F and C) significantly decreased after climatotherapy. *T*-scores for the positive mood subscale (V) increased in all sessions, but this increase was significant in only session 3. Furthermore, for all five negative mood subscales, *T*-scores from before the session gradually decreased throughout both spring and autumn.

**Table 8:** POMS *T*-score on six mood subscales before and after climatotherapy sessions at YF and GC (*n* = 43)

Spring							
	Mood subscale	T-A	D	A-H	V	F	C
Session 1 YF	Before session After session *t* *p* value Effect Size (Cohen’s *d*)	46.47 (44.08, 48.85) 39.72 (37.41, 42.04) 5.757 < 0.001 0.882	45.21 (43.48, 46.94) 41.67 (40.30, 43.05) 4.524 < 0.001 0.687	47.23 (45.37, 49.10) 41.05 (39.57, 42.52) 6.651 < 0.001 1.123	49.86 (47.20, 52.52) 51.30 (48.27, 54.33) − 1.380 0.175 − 0.154	45.56 (43.21, 47.91) 42.12 (39.84, 44.40) 2.581 0.013 0.457	47.67 (45.22, 50.12) 44.23 (42.11, 46.35) 4.271 < 0.001 0.455
Session 2 GC	Before session After session *t* *p* value Effect size (Cohen’s *d*)	44.42 (41.78, 47.06) 37.67 (36.52, 38.83) 5.800 < 0.001 0.918	43.79 (42.25, 45.33) 40.65 (40.04, 41.27) 4.617 < 0.001 0.731	44.84 (42.29, 47.38) 38.86 (38.21, 39.51) 4.606 < 0.001 0.989	50.12 (46.93, 53.31) 50.47 (47.55, 53.38) − 0.248 0.805 − 0.035	45.30 (43.05, 47.55) 39.53 (38.07, 41.00) 5.349 < 0.001 0.913	46.16 (43.65, 48.67) 41.86 (40.47, 43.25) 3.550 < 0.001 0.629
Session 3 YF	Before session After session *t* *p* value Effect size (Cohen’s *d*)	41.53 (39.54, 43.53) 37.09 (35.96, 38.22) 5.753 < 0.001 0.760	42.81 (41.37, 44.26) 40.95 (40.09, 41.82) 3.560 < 0.001 0.429	42.42 (40.73, 44.10) 38.93 (38.02, 39.84) 5.131 < 0.001 0.716	48.02 (45.38, 50.67) 51.47 (48.81, 54.12) − 3.520 < 0.001 − 0.400	43.58 (41.03, 46.13) 39.44 (38.01, 40.87) 3.695 0.001 0.580	44.58 (42.70, 46.46) 40.88 (39.54, 42.23) 5.986 < 0.001 0.646
Autumn							
	Mood subscale	T-A	D	A-H	V	F	C
Session 4 YF	Before session After session *t* *p* value Effect size (Cohen’s *d*)	44.81 (42.53, 47.10) 37.33 (36.20, 38.45) 6.738 < 0.001 1.228	45.28 (43.25, 47.31) 40.65 (39.95, 41.35) 5.256 < 0.001 0.795	45.84 (43.77, 47.90) 38.91 (38.19, 39.62) 6.948 < 0.001 1.300	50.00 (47.29, 52.71) 50.86 (47.72, 54.00) − 0.665 0.510 − 0.090	44.33 (42.51, 46.14) 40.58 (39.10, 42.06) 4.551 < 0.001 0.689	47.42 (44.76, 50.08) 41.30 (39.63, 42.98) 5.526 < 0.001 0.801
Session 5 GC	Before session After session *t* *p* value Effect size (Cohen’s *d*)	43.37 (41.07, 45.67) 37.84 (36.69, 38.99) 4.908 < 0.001 0.904	43.95 (42.02, 45.89) 40.88 (40.12, 41.64) 3.557 < 0.001 0.574	43.98 (41.75, 46.20) 38.91 (38.23, 39.59) 4.591 < 0.001 0.914	49.84 (47.04, 52.63) 49.98 (47.20, 52.75) − 0.126 0.900 − 0.015	44.33 (42.36, 46.29) 40.28 (39.09, 41.47) 3.726 < 0.001 0.762	46.47 (43.95, 48.98) 41.98 (40.47, 43.49) 4.107 < 0.001 0.632
Session 6 YF	Before session After session *t* *p* value Effect size (Cohen’s *d*)	42.56 (40.64, 44.47) 37.84 (36.71, 38.97) 5.660 < 0.001 0.873	44.07 (42.55, 45.59) 40.77 (40.05, 41.48) 4.396 < 0.001 0.829	42.95 (41.55, 44.36) 39.09 (38.25, 39.93) 5.051 < 0.001 1.017	49.63 (47.02, 52.24) 49.65 (46.63, 52.67) − 0.020 0.984 − 0.003	43.60 (41.32, 45.89) 40.19 (38.83, 41.54) 3.100 0.003 0.544	45.63 (43.69, 47.57) 41.84 (40.45, 43.22) 3.740 < 0.001 0.685

*T*-scores before and after the session are indicated as the mean (95% CI)

*CI* confidence interval, *T-A* Tension-Anxiety, *D* Depression-Dejection, *A-H* Anger-Hostility, *V* Vigour, *F* Fatigue, *C* Confusion

The POMS *T*-scores of all participants showed that in all sessions, the number of participants who improved to achieve the Iceberg profile (i.e. low scores on the five negative mood subscales and high scores on the one positive mood subscale V) (Morgan 1985) after the session exceeded the number who regressed from the Iceberg profile. The number of participants who improved to the Iceberg profile was 9, 10, 11, 12, 5 and 6 in sessions 1–6 (from spring to autumn), respectively, and including these, there were 20, 21, 24, 25, 18 and 19 participants who were Iceberg profile at the end of the sessions 1–6, respectively.

## Discussion

Our results showed that between the end of the spring sessions and the start of the autumn sessions, three participants in our programme developed a regular exercise habit. This finding is a good example of how our programme can facilitate behaviour change among participants. To increase physical activity, interventions that combine the five nudges ‘fun’, ‘easy’, ‘attractive’, ‘social’ and ‘timely’ with improved health literacy are effective (Takebayashi et al. 2022). Our climatotherapy programme also included five nudges and educational feedback to improve health literacy. On the other hand, the two women who stopped exercising regularly may have temporarily reduced their exercise to avoid the hot summer.

SBP, DBP and PR during each session varied to a greater extent at YF than at GC, suggesting that hilly paths at YF provided effective exercise. SBP and DBP after the fresh-air rest cure were higher at the first session in spring and the last session in autumn at YF than at the other sessions, possibly due to lower outdoor temperatures.

Furthermore, a comparison of the changes in SBP and DBP between before and after the climatic terrain cure (starting point and goal point) showed a greater decrease at YF than at GC. This finding suggests that increased blood flow and peripheral vasodilation, mainly in skeletal muscle, occurred on the YF path, which has a long uphill gradient and was selected with the intention of endurance training, compared to the GC path.

SBP and DBP were significantly higher after the fresh-air rest cure than after the climatic terrain cure at YF; the same trend was observed at GC, though there was no significant difference in SBP, only in DBP.

These results suggest that after the end of exercise, the body surface cooled down due to exposure to the open air, causing the subcutaneous capillaries to constrict. Blood pressure, particularly SBP, varied to a greater extent at YF, where the course was hilly and the outside air was cooler than at lowland GC, as expected. It is not surprising that the increased SBP during exercise returned toward normal at the end of exercise. On the other hand, it is suggested that resting in the open air in the forest environment induces physiological and psychological relaxation (Bielinis et al. 2019), which contributes to the SBP being lower than before the session. In a German study (Schuh 1997), the SBP of 98 inactive subjects decreased after 3 weeks of climatotherapy. Although our results represent a temporary reduction in SBP, continuous repetition of this programme may lead to an optimal SBP.

Among participants who did not take bradycardia-inducing antihypertensive drugs such as beta-blockers, percent PR_max_ was more than 10% higher at YF than at GC. Even in the session on the flat GC path, percent PR_max_ was 59.67%. This finding indicates that sessions at GC can maintain fitness for those without regular exercise habits or those who are unable to engage in high-intensity exercise.

The decrease in peripheral skin temperature during the climatic terrain cure suggests that our climatotherapy was properly implemented under conditions of a ‘slightly cool’ body surface (Schuh 1991). There are two possible reasons for the increase in peripheral skin temperature after the 20-min fresh-air rest cure. First, the body surface may not perspire while lying in open air with a ‘slightly cool’ body surface; thus, heat may not be lost through evaporative cooling. Second, there may have been increased peripheral blood flow due to relaxation and parasympathetic dominance (Chen et al. 2017). The greater reduction in peripheral skin temperature during the climatic terrain cure at YF compared with GC may reflect the cooler temperature at YF. Peripheral skin temperature is closely linked to the ambient temperature, and it is likely that a cool body surface was easier to achieve at YF in autumn, when temperatures during the sessions are lower than in spring.

sAMY is secreted by the sympathetic nervous-adrenal medullary system and by direct innervation of the sympathetic nervous system due to psychological and physical stress. The sAMY activity level after the session was lower compared to that before the session (and this decrease was significant in session 2), with the exception of session 3; however, sAMY measurements had a large SD deemed unsuitable for evaluation. In a study (Kobayashi, et al. 2010) that measured sAMY levels, SBP, PR and relaxation scale scores in outpatients before and after progressive muscle relaxation, guided imagery or autogenic training in a hospital setting, only sAMY levels showed no significant improvement. In our study, sAMY measurements were taken outdoors, which could easily have increased the variability of the measurements. Furthermore, staying in a relaxing environment and the fresh-air rest cure may have suppressed sympathetic activity, while physical exercise during the climatic terrain cure may have activated the sympathetic nervous system, making the sAMY data difficult to assess.

As exercise intensity increases, energy metabolism shifts from aerobic to glycolytic. Pyruvate produced with adenosine triphosphate (ATP) is incorporated into the citric acid cycle and metabolized into water and carbon dioxide. As exercise intensity increases, the excess pyruvate not incorporated into the citric acid cycle is reduced to lactate. Blood lactate levels remained almost unchanged, maintaining low levels at GC. After the climatic terrain cure at YF, which involves a long uphill path, blood lactate levels increased but did not exceed the OBLA, indicating that the exercise was performed within the aerobic range. Therefore, our programme was correctly implemented according to the aerobic endurance method of the climatic terrain cure.

At both YF and GC, scores on five of six POMS mood subscales were significantly improved after the climatotherapy session. Only the V subscale showed no significant change. Most of the subjects were elderly and retired; as expected, their mood was not as negative before the programme started. Despite this baseline, their mood improved significantly after the first session. This finding suggests that common factors at both climatotherapy sites, e.g. forest environment, spacious sites or enjoyable experiences, contributed to the improvement in their mood. The half-day sessions included in our climatotherapy programme took place in a forest environment, which meant that participants also experienced forest bathing through the five senses at the same time, meeting seven of the ten tips for forest bathing (Gallis and Shin 2020). And, during the fresh-air rest cure, participants were very relaxed, and some fell asleep. This relaxation could be one of the reasons why the V scores did not exhibit significant changes.

Furthermore, the scores on all five negative mood subscales before the start of the session gradually decreased in both spring and autumn, suggesting that the mood-improving effects of climatotherapy still remained after 1 week.

More than 60% of the participants had an exercise habit before the start of the programme, but the number of participants whose mood improved to the Iceberg profile (Morgan 1985), which is associated with positive mental states and athletic peak performance, was particularly high in the second and third spring sessions and the first autumn session. At the climatotherapy sites in late autumn, the scenery of dull-coloured trees and fallen leaves is widespread and calming, which may be one of the reasons why the V scores of participants did not increase. As a result, few participants improved to the Iceberg profile in late autumn sessions, and some participants even deteriorated from the Iceberg profile. On the other hand, early spring and late autumn are suitable for climatotherapy, which focuses on cool body surfaces, because the temperatures are cooler and the body surface is easily cooled during exercise.

Climatic terrain cures aim to train the body by applying exercise loads under the condition of a cool body surface, but in addition to this, even flat pathways and tree-rich environments can improve mood (Kanayama et al. 2017). Although it is difficult to perform aerobic exercise effectively on flat pathways, the GC site was considered suitable for people without regular exercise habits or elderly people to start exercising. However, some participants exercised regularly before starting the programme. For such people, the exercise intensity of the climatic terrain cure at GC was insufficient. In general, people who exercise regularly are more likely to participate in such programmes. More than 70% of the subjects over 60 years had regular exercise habits, a higher percentage than that of the national statistics. On the other hand, just over 10% of subjects under 59 years old had regular exercise habits, which was a lower percentage than that of the national data. The large difference in the percentage of regular exercise habits between working age and retired people suggests that our programme can be made more useful to a wider range of people if the pace of walking is tailored to participants’ exercise habits and fitness levels.

There are several limitations. (1) Our study did not take a randomised controlled design and there was no control group with no intervention at all. (2) Since the programme ended in the autumn, no follow-up studies have been carried out. (3) Weather data were only collected at the site where the climatotherapy sessions took place. Thus, the climatic environment at the two sites, YF and GC, cannot be fully compared. (4) There were more participants with regular exercise habits than those with inactivity, the most appropriate indication. One possible reason for this is that more than 80% of the participants were retired people with more time on their hands; another is that people who are more interested in their health are more likely to participate in such programmes.

Future research should be conducted to address these limitations.
